# The circadian rhythm in intervertebral disc degeneration: an autophagy connection

**DOI:** 10.1038/s12276-019-0372-6

**Published:** 2020-01-27

**Authors:** Tai-Wei Zhang, Ze-Fang Li, Jian Dong, Li-Bo Jiang

**Affiliations:** Department of Orthopedic Surgery, Zhongshan Hospital, Fudan University, Shanghai, 200032 China

**Keywords:** Macroautophagy, Mechanisms of disease, Senescence

## Abstract

There is one circadian clock in the central nervous system and another in the peripheral organs, and the latter is driven by an autoregulatory molecular clock composed of several core clock genes. The height, water content, osmotic pressure and mechanical characteristics of intervertebral discs (IVDs) have been demonstrated to exhibit a circadian rhythm (CR). Recently, a molecular clock has been shown to exist in IVDs, abolition of which can lead to stress in nucleus pulposus cells (NPCs), contributing to intervertebral disc degeneration (IDD). Autophagy is a fundamental cellular process in eukaryotes and is essential for individual cells or organs to respond and adapt to changing environments; it has also been demonstrated to occur in human NPCs. Increasing evidence supports the hypothesis that autophagy is associated with CR. Thus, we review the connection between CR and autophagy and the roles of these mechanisms in IDD.

## Disc degeneration: Damage from disrupted cellular circadian rhythms

Disruption of molecular activities that follow a 24-hour cycle, known as a circadian rhythm, can promote the degeneration of the discs between vertebrae by affecting the natural degradation of cellular components known as autophagy. Zhang Taiwei and colleagues at Fudan University in Shanghai, China, review the role of autophagy in mediating a connection between the circadian rhythm within intervertebral disc cells and disc degeneration. Some evidence suggests that while normal autophagy maintains discs, dysfunction in the circadian rhythm within disc cells can initiate damaging excessive autophagy. The influences leading to such dysfunction include nutritional status and behavioural factors including night-shift work and otherwise disrupted sleep patterns. Contradictory evidence, however, suggests that further investigation is needed to clarify the molecular mechanisms that link circadian rhythms, autophagy and disc degeneration.

## Introduction

Approximately 2/3 of adults suffer from lower back pain (LBP), for which age-related degenerative processes in intervertebral discs (IVDs) and intervertebral disc herniation are the most common reasons^1^. Each IVD is composed of a cartilage end-plate (CEP), a nucleus pulposus (NP) and an annulus fibrosus (AF)^2^. All three of these components are involved in intervertebral disc degeneration (IDD)^3,4^. It has long been known that diurnal shifts in mechanical loading lead to osmotic pressure changes, and these two types of alterations together with other microenvironmental factors stimulate nucleus pulposus cells (NPCs), contributing to the stress of NPCs^5–7^. Recently, an autoregulating circadian rhythm (CR) was identified in IVDs, the abolition of which led to IDD^8^. The abolition of CR is also observed in osteoarthritis (OA) cartilage, and the degeneration of cartilage can be induced by BMAL1 or REV-ERBα knockdown with loss of TGF-β signaling^9^.

Circadian clocks are time-measuring devices that are present in most light-sensitive organisms. The central nervous system interacts with surrounding tissues with a 24-h oscillation period, controlling nutrient metabolism, energy balance, redox status and organismal behavior and maintaining homeostasis through complicated pathways^10^. Autophagy is responsible for a significant connection between CR and homeostasis, as will be discussed below.

Autophagy (macroautophagy) is a fundamental cellular process in eukaryotes that is essential for individual cells or organs to respond and adapt to changing environments in which cells digest themselves to generate life-supporting substances^11^. Autophagy plays prominent roles in determining the life spans of many model organisms and in pathological processes in many organs^11^. Additionally, autophagy maintains the homeostasis, inhibits the apoptosis and prevents the senescence of NPCs^12^.

Recently, evidence of an interaction between CR and autophagy has emerged. Many genes involved in different steps of the autophagy pathway, such as Atg14, UNC51-like kinase 1 (Ulk1), GABA(A) receptor-associated protein-like 1 (Gabarapl1), microtubule-associated protein 1 light chain 3 B (LC3B), and BCL2/adenovirus E1B-interacting protein 3 (Bnip3), have been found to be associated with CR^13^. In this review, we discuss how autophagy is connected to IDD and CR and what we can do as a next step to clarify the mechanisms by which CR and autophagy are involved in IDD by summarizing evidence from both in vivo and in vitro studies.

## Overview of the molecular circadian clock

There are two kinds of circadian clocks: one in the central nervous system and another in surrounding tissues. The former is controlled by suprachiasmatic nucleus neurons following light stimulation, and the latter is controlled in every cell by the expression of clock genes^14^. The cell-autonomous molecular clock in mammals is generated by two interlocking transcription/translation feedback loops (TTFLs) that function together to produce robust 24 h rhythms of gene expression^15^. The core TTFL is driven by four integral clock proteins, including two activators (circadian locomotor output cycles kaput (CLOCK) and brain and muscle ARNT-like protein-1 (BMAL1, also known as ARNTL)) and two repressors (Period (Per) and cryptochrome (Cry)) as well as by kinases and phosphatases that regulate the phosphorylation (P) and thereby the localization and stability of these integral clock proteins (kinases: CKIα, CKIδ, and CKIɛ; phosphatases PP1, PP5)^15^. The fundamental principle of the molecular clock in mammals relies on a transcriptional activator inducing the transcription of a repressor, which results in the accumulation of the latter over time until it reaches a sufficient level to repress its own activation^16^. The basic helix-loop-helix PER-ARNT-SIM (bHLH-PAS) proteins CLOCK and BMAL1 are the primary transcriptional activators within the circadian clock mechanism of mammals^15^. The CLOCK and BMAL1 transcription factors act as heterodimers to activate the Per and Cry gene families through interaction with E-box regulatory sequences^16^. As a result, the Per and Cry protein products form heterotypic complexes that accumulate over time in the cytoplasm^16^. The levels of Per/Cry complexes increase until they reach a threshold, marking the beginning of the repressive phase, in which the Per/Cry complexes translocate back to the nucleus and repress CLOCK/BMAL1 transactivation^15^. REV-ERBα/β and retinoic acid-related orphan receptor α (RORα) are major regulators of cyclic transcription within the positive limb of the mammalian circadian oscillator, although their roles are nonessential^15^. Posttranslational regulation mechanisms, such as phosphorylation, acetylation, methylation, sumoylation, and glycosylation, then modulate protein turnover, intracellular localization, and DNA-binding affinity^16^. CR then regulates NAD^+^ biosynthesis, amino acid and carbohydrate metabolic pathways, and nucleotide biosynthesis, thus contributing to glucose homeostasis, energy homeostasis, hematopoietic cell homeostasis, epidermal stem cell homeostasis, and many other vital activities^17,18^.

## The circadian rhythm in intervertebral discs

Many characteristics of the relationship between IVDs and CR have long been known. Body height changes during the day^19^. Over 50% of the height loss in a day occurs within the first hour of rising, and 80% occurs within 3 h of rising; the rate of creep decelerates throughout the remainder of the waking day^19^. One of the reasons for these changes is pressure-dependent fluid shifting in the IVDs^20^. When an IVD is subjected to a diurnal cycle involving 16 h of loading followed by an 8-h recovery period, the osmotic pressure of the nucleus becomes much higher than that of the annulus, which is why fluid is drawn back into the disc after the end of loading^5^. A decrease in osmotic pressure in the central nucleus region through degeneration leads to an inability to draw fluid back into the disc^5^. The range of lumbar flexion is increased during the day compared to that during the night and increases after creep, suggesting that forward bending movements subject the lumbar spine to higher bending stresses in the early morning than later in the day; this increase is ~300% for the discs and 80% for the ligaments of the neural arch^21^. Discs appear to be very sensitive to their prevailing osmotic environment (at least in vitro), which has a powerful effect on matrix synthesis and, hence, ultimately on the structure and composition of the discs during each diurnal cycle^6^. Penetration through the end-plate increases with diurnal loading^22^.

**Fig. 1:**
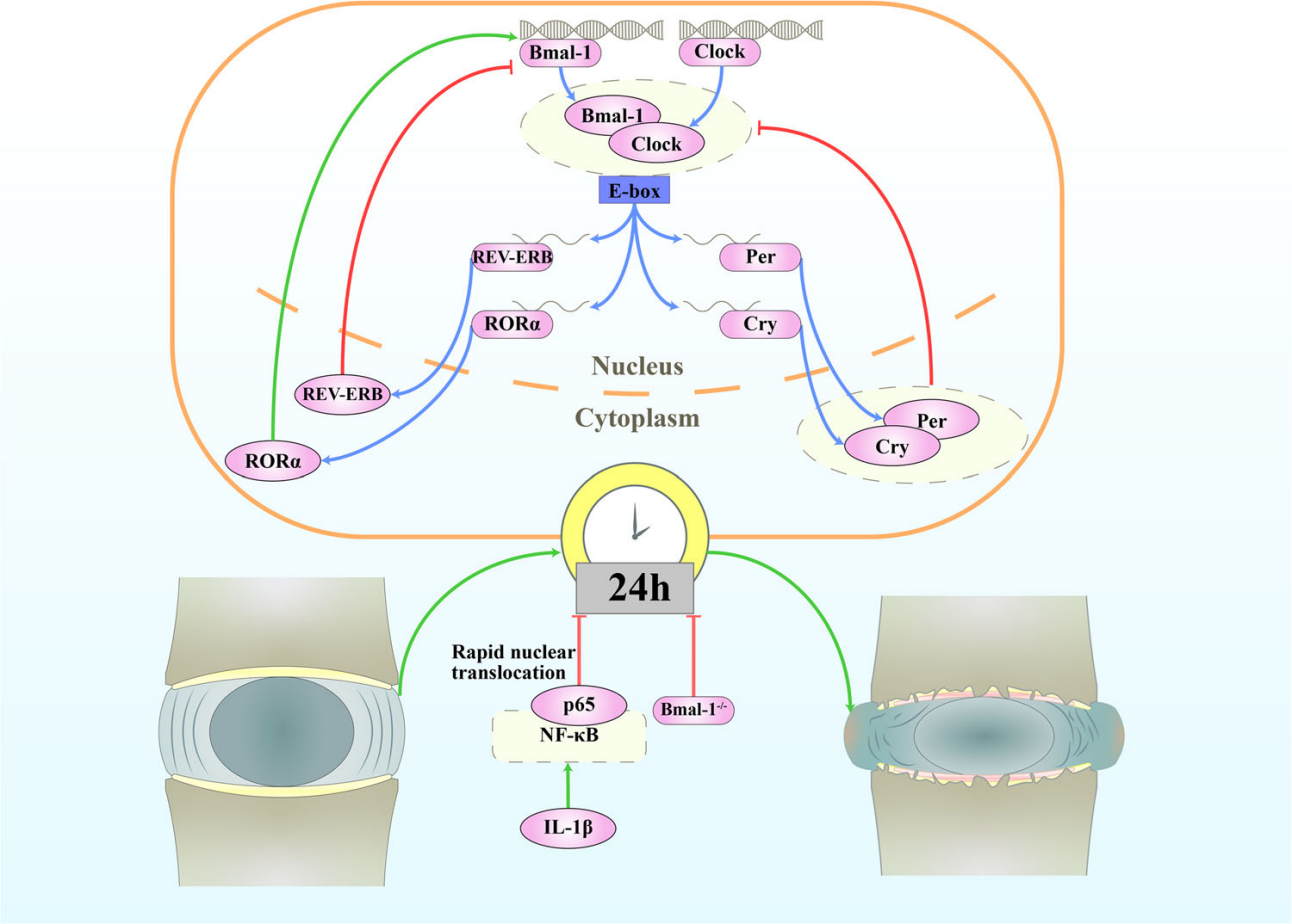
The circadian rhythm in intervertebral disc degeneration. The amplitude of the oscillations in IVDs from aged mice is attenuated, with decreased expression of the core circadian transcription factors BMAL1 and CLOCK. Knockout of Bmal1 in mice IVD cells leads to the widespread degeneration of lumbar IVDs. Bone bridges appear within the growth plate, the CEP is almost completely replaced by bone, and the height of the disc is significantly reduced. Disorganization of the outer annulus structure and signs of fibrosis (with organized collagen bundles) appear at the periphery of the IVDs. IL-1β causes the rapid nuclear translocation of p65 in both AF and NP cells and leads to a loss of pacemaking properties of individual cells.

The CR of IVDs is dampened with aging in mice and can be abolished by treatment with interleukin (IL)-1β^8^. The genetic disruption of the mouse IVD molecular clock, specifically through the disruption of BMAL1, predisposes mice to IDD^8^. This finding suggests that the disruption of CR may be a risk factor for degenerative IVD disease and LBP (Fig. 1)^8^. Some upstream factors and downstream pathways that interact with CR in IVDs have also been found. Passive cigarette smoking changes the CR of clock genes in rat IVDs, with most genes showing a phase shift of −6 to −9 h and some clock-related genes showing abolished oscillation in the NP^7^. BMAL1 and RORα regulate hypoxia-inducible factor (HIF)-1 activity in NPCs and play important roles in the overall adaptation of NPCs to their hypoxic niche; the dysregulation of these proteins affects normal tissue homeostasis and function^23^. HIFs coordinate cellular adaptations to low-oxygen stress by regulating transcriptional programs in erythropoiesis, angiogenesis, and metabolism^24^.

## Overview of autophagy

The canonical process of autophagosome formation, which is evolutionarily conserved, involves the stages of initiation, nucleation, elongation and closure, recycling and degradation^25^. It is usually promoted by the direct activation of Ulk1 through Ulk1 phosphorylation by adenosine 5′-monophosphate (AMP)-activated protein kinase (AMPK)^26^. The mammalian target of rapamycin kinase complex 1 (mTORC1) is incorporated into the Ulk1–Atg13–FAK family kinase-interacting protein of 200 kDa (FIP200) complex, and mammalian target of rapamycin (mTOR) kinase phosphorylates Ulk1 Ser 757, disrupting the interaction between Ulk1 and AMPK and suppressing autophagy directly^26,27^. AMPK also phosphorylates Raptor to suppress the inhibitory effect of mTORC1 on the Ulk1 autophagic complex^28^.

When autophagy is induced, Ulk1 phosphorylates AMBRA1, releases the Beclin1 complex and produces phosphatidylinositol 3-phosphate (PtdIns3P or PI3P) from dynein^29–31^. PI3P is recognized by WD-repeat protein Interacting with Phosphoinositides (WIPI) proteins in the endoplasmic reticulum and is rapidly recruited to the site of autophagosome formation^29^. Beclin1 forms a complex with ER-associated Bcl-2 under nutrient-rich conditions and is released upon the phosphorylation of Bcl-2 by c-Jun N-terminal kinase 1 (JNK 1)^32^.

Atg8 (in yeast)/microtubule-associated protein 1 light chain 3 (LC3) is cleaved at its C terminus by ATG4 to generate cytosolic LC3-I with a C-terminal glycine residue, which is conjugated to phosphatidylethanolamine (PE) in a reaction that requires ATG7 and the E2-like enzyme ATG3^33^. The LC3-binding protein p62 is a specific substrate for autophagy, and the Atg12–Atg5–Atg16L1 complex is also required for the formation of the covalent bond between LC3 and PE^32^. The lipidated form of LC3 (LC3-II) is attached to both faces of the phagophore membrane but is ultimately removed from the autophagosome outer membrane; this removal is followed by the fusion of the autophagosome with a late endosome/lysosome^33^. Autophagy occurs at low basal levels in virtually all cells to perform homeostatic functions such as protein and organelle turnover^34^. Autophagy is upregulated under conditions of starvation, growth factor withdrawal, high bioenergetic demands, oxidative stress, infection, or protein aggregate accumulation^34^.

## Autophagy in intervertebral discs

**Fig. 2:**
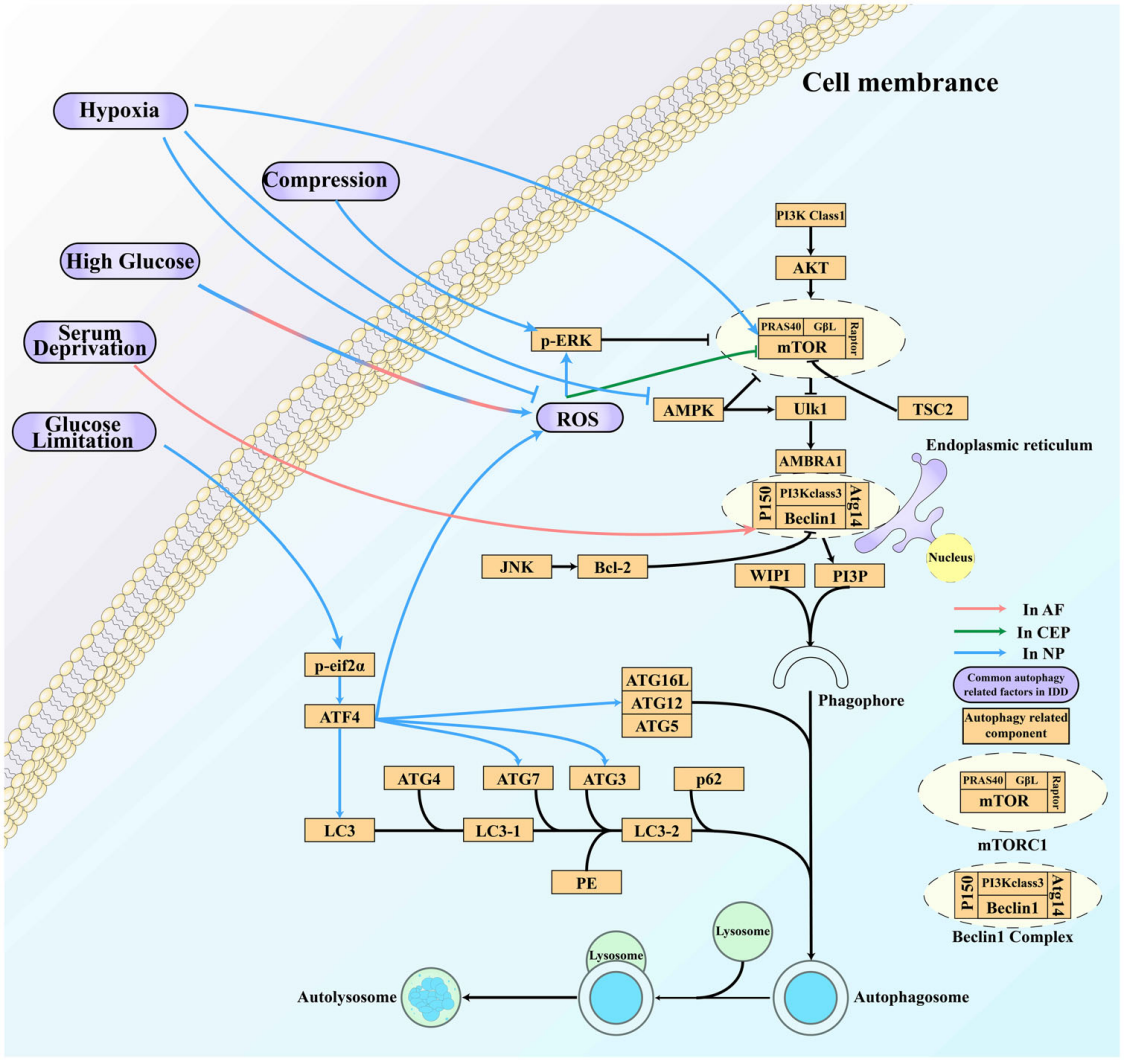
Autophagy in intervertebral disc degeneration. Compression, high glucose levels, serum deprivation, and glucose limitation are common stimuli that activate autophagy in IVDs through different pathways and play various roles in IDD. In contrast, hypoxia inhibits autophagy through the classic autophagy pathway in NPCs. ROS are factors involved in high glucose-, serum deprivation- and hypoxia-dependent autophagy regulation that downregulate mTOR signaling.

The end-plate chondrocytes (EPCs) of cervical spondylosis patients show decreased autophagy compared with those of fracture and dislocation patients^35^. During the aging process, the expression of the autophagy-related genes LC3 and Beclin-1 significantly decreases with the decreasing activity of EPCs^36^. In contrast, autophagy has been demonstrated to occur and even to increase during normal aging in NPCs^37^. Autophagy has also been shown to be upregulated in AF cells (AFCs) in IDD patients^38^. The current findings regarding the conditions influencing autophagy in IVDs and their associated pathways and effects are summarized in the Supplementary information (Fig. 2).

Among these factors, tumor necrosis factor-α (TNF-α) and IL-1β regulate autophagy^39,40^. However, some studies have shown that neither TNF-α nor IL-1β can regulate autophagy in rat NPCs and that IL-1β alone cannot induce autophagy in AFCs^41,42^. In rats, IL-1β does not induce autophagy in AFCs by itself but augments the autophagy induced by serum deprivation; such autophagy may contribute to delays in apoptosis and IDD^42^. Hyperosmotic stress may activate the autophagy of NPCs via the Ca^2+^-dependent AMPK/mTOR pathway^43^. However, this is still a controversial theory^44^. Specifically, mechanical loading is one of the factors that causes IDD^45^.

Although the mTOR pathway is involved in many of the conditions listed in the Supplementary information, the pharmacological inhibition of only mTORC1, and not mTORC2, protects against human disc apoptosis, senescence, and extracellular matrix catabolism through Akt and autophagy induction^46^.

## The circadian rhythm of autophagy

The presence of autophagic vacuoles and the atrophy of the liver was found to follow a diurnal pattern in meal-fed rats in comparison with nutrient-deprived controls using electron microscopy^47^. This was the first evidence of a link between autophagy and circadian regulation, which was established in the early 1970s^47^. Additional evidence of autophagy regulation by CR in the kidney, heart, liver, brain, and retina has been found via electron microscopy, autophagic flux measurements and fluorescence measurements, not only in mammals but also in Drosophila and zebrafish^13,48–59^.

The autophagic rhythm varies from tissue to tissue. In the convoluted tubules of the kidney, analyses of the number of autophagic vacuoles per area unit and the total amounts of segregated material have shown that the minimum values occur during the night, while the maximum values occur during the day^58^. The volume and numeric density of autophagic vacuoles in the heart peak during the late-light phase and later decline toward the early dark period^53^. Autophagy flux has been found to reach its peak in the afternoon, rapidly decrease at night and rise again throughout the light phase in the mouse liver^13^. The daily rhythms of liver autophagy might not directly arise from the molecular circadian oscillator but may instead be indirectly coupled to the molecular clock via feeding or other normally circadian-gated behaviors^56^. This evidence suggests that autophagy can be determined by the internal environment of a specific organ regardless of the regulation of the central nervous system.

## The circadian rhythm regulates autophagy

Integration between CR and behavior or the environment is vital for normal physiological processes. The feeding/fasting rhythm has been found to regulate autophagy in muscle, adipose and liver tissue and to play a protective role in metabolism and life span^60^. Intermittent fasting (IF) has been shown to restore autophagic function, thereby preserving organelle quality^61^. The autophagy-associated protein LC3-II has been shown to exhibit a CR of its protein expression in the hippocampus, which is dampened by sleep fragmentation (SF).^51^ However, recovery sleep did not return LC3 expression to the basal oscillating level^51^. The basal levels of autophagy within the outer retina change in a dynamic manner during the course of the day and night and are regulated by the circadian light input^49,52,62^. In the neural retina, cyclic illumination is one of the factors that activates autophagy^63^.

Recently, it has been demonstrated that autophagy levels are controlled by several clock genes, especially Period2 (Per2) and BMAL1. Per2 in the liver functions as a scaffold protein to tether tuberous sclerosis complex 1 (TSC1), Raptor, and mTOR together to specifically suppress the activity of the mTORC1 complex^64^. Knockdown of Per2 downregulates autophagy levels while leaving core clock oscillations largely intact^65^. Knockdown of Per2 also reduces cellular levels of the Ulk1 protein without affecting Ulk1 mRNA levels, consistent with the rhythmic Ulk1 protein levels and nonrhythmic Ulk1 mRNA levels in these cells^65^. The transient overexpression of Per2 results in the downregulation of the PI3K Class 1/Akt pathway in vitro and increases autophagic flux^65,66^. Tor, ATG5 and ATG7 exhibit rhythmic expression in the brains of wild-type flies under day/night conditions (LD, 12:12), which is abolished in Per1 clock mutants^59^. C/EBPβ is a transcription factor that is regulated by CR and induces the expression of autophagy genes such as Ulk1, Gabarapl1, LC3B, and Bnip3 and the degradation of proteins^13^. However, its expression and the expression of the autophagy-related genes LC3B, GABA(A) receptor-associated protein a (Gabarapa), ATPase H^+^-transporting lysosomal V1 subunit D (Atp6v1d), ATG4a, ATG4d, Beclin1, Bnip3, Ulk1a, and Ulk1b are upregulated without rhythmicity in Per1b mutant fish^50^. These findings suggest the indispensable role of Per in maintaining the rhythmicity of autophagy.

In the heart, BMAL1 protects cardiomyocytes under hyperglycemic conditions by inducing autophagy through mTORC1 signaling downregulation^67^. BMAL1^−/−^ mice exhibit increased LC3A and decreased p62 levels in muscle, whereas Beclin-1 levels are unchanged^68^. AMPK activation has been indicated to be involved in the regulation of BMAL1 in autophagy^68^. Cardiomyocyte-specific BMAL1 knockout (CBK) and cardiomyocyte-specific Clock mutant (CCM) mice exhibit hyperactivation of the PI3K Class 1/Akt/mTOR signaling axis, which likely contributes to attenuation of autophagy and the augmentation of protein synthesis^69^. Thus, the expression of BMAL1 may be associated with the activation of autophagy.

In vitro, REV-ERB agonism blocks autophagy and induces apoptosis^70^. In vivo, REV-ERB agonism has been found to downregulate the mRNA and protein levels of Ulk3, Ulk1, Beclin1, and ATG7 and to significantly improve survival in two glioblastoma models^70^. In zebrafish, REV-ERBα has been found to bind directly to the ROR-responsive element (RORE) sites in the Ulk1a promoter, thereby repressing Ulk1a in vivo, and another autophagy-related gene, Atp6v1d, is a direct target of REV-ERBα^52^. However, it has also been shown that REV-ERBβ does not seem to be directly involved in autophagy regulation but likely acts as a cytoprotective factor downstream of a blockade of autophagy^71^.

Other core clock proteins also show the ability to influence autophagy. AMPK activity and nuclear localization are rhythmic and are inversely correlated with Cry1 nuclear protein abundance^72^. The rhythmic regulator CLOCK may potentially affect tumor cell resistance to cisplatin by inducing autophagy^73^. ATG14 gene expression, which is controlled by CLOCK/BMAL1 binding to its E-box, also exhibits a CR^74^

## Autophagy regulates the circadian rhythm

Although there is relatively less evidence that autophagy regulates CR than that CR regulates autophagy, findings related to the core components of autophagy and CR are included in such evidence. The silencing of Tor in Per-expressing cells shortens the period of the locomotor activity rhythm of flies^59^. The stimulation of AMPK destabilizes cryptochromes and alters CR, and mice in which the AMPK pathway is genetically disrupted show alterations in their peripheral clocks^72^. The circadian proteins BMAL1, CLOCK, REV-ERBα, and Cry1 are lysosomal targets, and the selective autophagic degradation of Cry1 occurs in a diurnal window when rodents rely on gluconeogenesis, suggesting that Cry1 degradation is time imprinted for the maintenance of blood glucose^75^. In addition, high-fat feeding accelerates autophagic Cry1 degradation and contributes to obesity-associated hyperglycemia^75^. A CLOCK mutant attenuates BMAL1 degradation through both proteasomal and autophagic pathways under high-fat diet feeding, providing evidence of autophagic regulation of a core clock component^76^.

## Indirect regulation between autophagy and the circadian rhythm in intervertebral discs

The circadian regulator CLOCK is a histone acetyltransferase (HAT) that also acetylates a nonhistone substrate: its own partner, BMAL1^77^. Both nonhistone acetyltransferase and HAT activities are essential to the circadian rhythmicity and activation of clock genes^77^. Sirt1 is a circadian deacetylase for core clock components^78^. The NAD(+)-dependent enzyme Sirt1, which functions as a histone deacetylase whose activity is also regulated by the redox states of NAD cofactors, counteracts the activity of CLOCK^77^. Additionally, Sirt1 binds to CLOCK-BMAL1 and Per2 in a circadian manner and supports the deacetylation and degradation of Per2^79^. In the absence of Sirt1, constitutively high protein levels of Per2 may lead to the repression of Per1, Per2, Cry1, and retinoic acid-related orphan receptor γ (RORγ) mRNA expression^79^. On the other hand, Sirt1 may activate autophagy through the Sirt1-LKB1-AMPK pathway and by deacetylating ATG5, ATG7, LC3, and tuberous sclerosis complex 2 (TSC2), a component of the mTOR inhibitory complex upstream of mTORC1^80–83^. Taken together, these findings suggest that Sirt1 serves as a link between autophagy and CR and between the redox state and the circadian clock.

Melatonin, an endocrine hormone synthesized and secreted by the pineal gland in the brain that helps to maintain CR, significantly enhances protective effects in different systems, including the central nervous, cardiovascular, gastrointestinal and endocrine systems, through the enhancement or inhibition of the autophagy process^84^. It is believed that oxidative stress can activate autophagy; for this reason, the antioxidant activity of melatonin could account for its inhibitory effects on autophagy^85^. If so, melatonin may affect mechanisms that stimulate autophagy, rather than affecting the process itself^85^. Melatonin seems to inhibit autophagy triggered by either mTOR activation or JNK/Bcl-2/Beclin1 pathway signaling^86,87^. Cyclosporine A is known to induce autophagy via ER stress^88^. Melatonin suppresses cyclosporine-induced autophagy in rat pituitary GH3 cells through the MAPK/ERK pathway, an effect that is due either totally or in part to the antioxidant properties of melatonin^85,88^. Deficiency of the nuclear melatonin receptor RORα aggravates autophagy dysfunction in diabetic hearts and myocardial ischemia/reperfusion injury in mice^89–91^. However, in regimen 1-treated N2a/APP cells, only a slight increase in cellular autophagy has been found using flow cytometry, and there is no significant alteration in the expression of the autophagy-associated markers Beclin-1 and LC3-I/II^92^. These results suggest that autophagy may play a negligible role in the beneficial effects of caffeine, melatonin, and coffee on AD^92^.

The circadian regulation of C/EBPβ and the autophagy disruption observed in mice lacking a functional liver clock suggest that C/EBPβ is a key factor that links autophagy to the biological clock and maintains nutrient homeostasis throughout light/dark cycles^13^. In zebrafish, a CLOCK-BMAL1 heterodimer binds to E-boxes to regulate the transcription of C/EBPβ, which in turn controls the transcription of autophagy-related genes indirectly^50^.

Fus1, a tumor suppressor protein residing in mitochondria, maintains mitochondrial homeostasis and is highly expressed in the brain^93^. One study revealed that KO mice showed sleep/wake disturbances compared to WT mice and that the autophagy marker LC3-II was decreased in both the olfactory bulbs and hippocampi, suggesting an early onset of autophagy dysregulation in Fus1 KO mice^93^. Heme oxygenase (Ho), whose silencing results in the downregulation of autophagy-related genes in both light and dark phases, is expressed in a circadian manner^94^. FoxOs are tightly controlled by fasting/feeding cycles and can upregulate the expression of ATG14^74^. Casein kinase 1α (CK1α) exhibits dual functions in autophagy regulation^95^. CK1α-mediated phosphorylation stimulates the degradation of Per1, suggesting a function in CR^95^.

The expression of the core clock genes Per2 and REV-ERBα is increased after weight loss^96^. Clock gene expression levels and their weight loss-induced changes are tightly correlated with each other and with the expression of genes involved in autophagy (LC3A and LC3B)^96^. Folic acid deprivation increases autophagic activity in hippocampal neuron cells, an effect that is associated with the activation of autophagy- and circadian-related genes^97^.

**Fig. 3:**
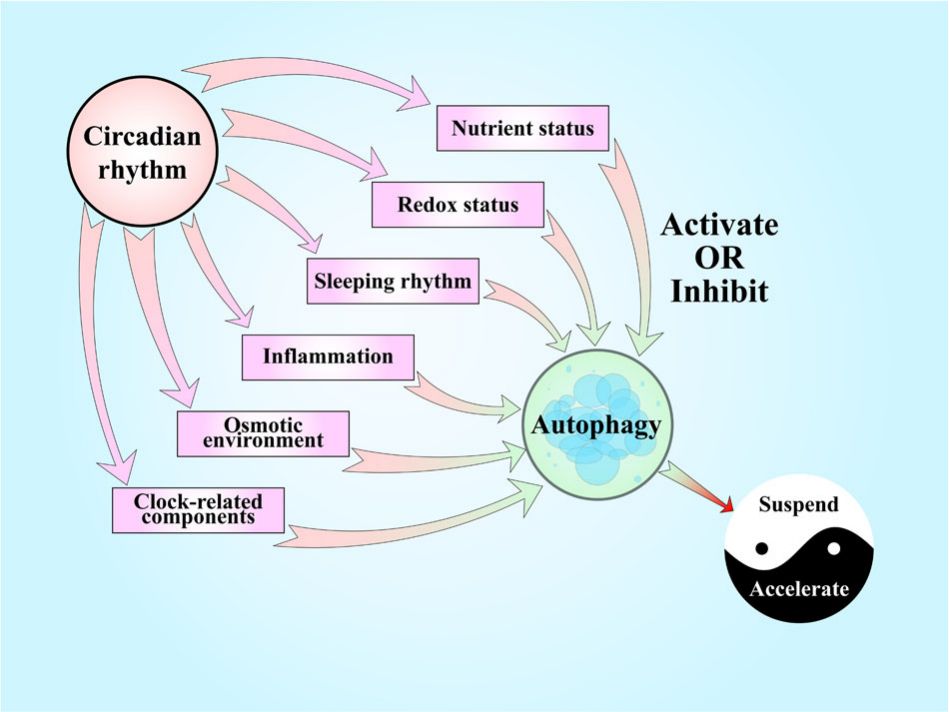
The link between autophagy and the circadian rhythm in the regulation of intervertebral disc degeneration. Nutrient status, redox status, sleep rhythms, inflammation, and the osmotic environment are common factors affecting IVDs that show circadian rhythms in normal conditions and present close connections with autophagy. The intensity and duration of these stimuli determine the consequences of autophagy, affecting IDD. Clock-related components have recently been found to play protective roles in IVDs have been shown to affect autophagy in other organs.

In the brain, acrylamide (ACR), a chronic neurotoxin, substantially attenuates spontaneous alternation. ACR dampens the oscillatory amplitudes of clock genes (BMAL1, Cry2 and REV-ERBβ) or causes a phase shift in clock genes (CLOCK, Per2 and REV-ERBα) and weakens the amplitude of Sirt1 oscillations. In addition, ACR increases the number of autophagic structures only in the night phase, which may cause nerve cell damage and apoptosis, ultimately leading to cognitive impairment (Fig. 3)^98^.

## Discussion and prospects

To the best of our knowledge, there are few studies linking autophagy and CR in IVDs. However, the findings reported to date provide some insights and clues that CR induces IDD at least partially in an autophagic manner. Nutrient status, oxidative stress, inflammation, the osmotic environment and mechanical loading are the most common factors that induce changes in autophagy and CR in IVDs. Considering the great deal of evidence regarding nutritional status^42,56,60,61,74–76^, we are also particularly interested in this field. As we described above, a periodic lack of nutrition favors the molecular CR, autophagy and their interaction, while a lack of nutritional oscillation, as observed in the context of diabetes, leads to dysfunction of autophagy^60,61,74^. Limited nutritional deprivation can activate autophagy and lead to cell renewal in IVDs^99–101^. Thus, we can infer that nutrition is an incentive for CR, leading to the further regulation of autophagy. Redox status is also a prevalent factor involved in many metabolic processes. Not only H_2_O_2_ but also an unsuitable nutrient status induces autophagy in an ROS-dependent manner in IVDs^101^. Sirt1 and melatonin are bridges between redox status and autophagy; one of them regulates clock-related molecules, while the other is regulated by CR^77,84^. It has long been known that night shift work is a significant risk factor for the development of IDD and its progression^102^. SF has been indicated to blunt CR and damage the expression of autophagy-related proteins in the CNS, perhaps permanently^51^. Given all of these findings, we can ask whether sleep rhythms regulate autophagy in IVDs. Night shift work also involves abnormal loading/resting cycles^102^. In these cycles, IVDs undergo changes in the mechanical loading and activation of autophagy, which can result in IDD^45^. As the most recent research has shown, inflammation is a significant factor in IDD, as IL-1β abolishes the normal molecular functions of clock-related components, participates in multiple pathological processes during disc degeneration (including inflammatory responses, matrix destruction, angiogenesis and innervation, apoptosis, oxidative stress and cellular senescence), and activates protective autophagy in IVDs^103^. However, despite this contradictory evidence, one thing is clear: clock dysfunction is a key factor associated with inflammation-related autophagy in IDD. The water content varies in IVDs during the loading/resting cycle together with changes in the osmotic environment^5,20,22^. The osmotic environment is also associated with autophagy in IVDs^43^. Whether this phenomenon affects the molecular CR in IVDs could be investigated in the future. Given the core circadian regulation observed in IVDs and the autophagy regulation mediated by components, such as Per^65^, BMAL1^67,68^ and REV-ERB^70^ in other models, we can hypothesize that there is a fairly close interaction between CR and autophagy.

We can infer that limited autophagy coordinated by CR protects IVDs from degeneration, while excessive autophagy and autophagic dysfunction induced by abnormal CRs under different clinical conditions accelerate IDD. Since an intrinsic CR has already been found in IVDs, further research should investigate the molecular mechanism by which CR influences autophagy in the process of IDD.

## Supplementary information

Table S1
