# Supplementary material for: The circadian rhythm in intervertebral disc degeneration: an autophagy connection
Source: Exp Mol Med. 2020 Jan 27;52(1):31–40. doi: 10.1038/s12276-019-0372-6 (PMC7000407; doi:10.1038/s12276-019-0372-6)
Supplement: Supplementary file 1 — Table S1 [file 12276_2019_372_MOESM1_ESM.doc]

**supplementary information**

**Table S1** Summary of the current findings regarding conditions influencing autophagy in IVDs

| Condition/factor | Pathway | Activate/Inhibit | Protective/harmful | Reference |
| --- | --- | --- | --- | --- |
| In CEP | | | | |
| H2O2 | MTOR | Activate | Protective | 1 |
| Melatonin | Sirt1 | Activate | Protective | 2 |
| Short-term Intermittent cyclic mechanical tension (ICMT) | - | Activate | Protective | 3 |
| Long-term ICMT | - | Inhibit | Harmful | 3 |
| In NP | | | | |
| High glucose | Adapt response | Activate | Protective | 4 |
|  | ROS | Activate | Protective? | 5,6 |
| Short-term Glucose limitation | eukaryotic translation initiation factor 2 (eIF2α)/activating transcription factor 4 (ATF4) | Activate | Protective | 7,8 |
| Long-term Glucose limitation | P53 | Activate | Harmful | 9 |
| Short-term Serum deprivation | MTOR-independent | Activate | Protective | 10 |
| Long-term Serum deprivation |  |  | Harmful |  |
| Hypoxia | Restricting ROS generation & AMPK/mTOR/HIF-1α | Inhibit | Protective | 10 |
|  | NF-κB/REDD1 | Inhibit | Protective | 11 |
|  | MTOR/HIF1-independent | Activate | Unknown | 12 |
| H_2_O_2_ | Erk/mTOR | Activate | Protective | 13 |
| Islet amyloid polypeptide (IAPP) | PI3K Class1/Akt/ mTOR | Activate | Protective | 14,15 |
| Sirt1 | - | Activate | Protective | 16,17 |
| Sirt6 | MTOR | Activate | Protective | 18 |
| Sestrins (sesn) | MTOR | Activate | Protective | 19 |
| Transcription factor EB (TFEB) | Inducing lysosome biogenesis/ promoting autophagosome formation/ fusion with lysosome | Activate | Protective | 20 |
| TNF‐α | PERK/eif2α/ mTORC1 | Activate | Protective | 21 |
| IL-1β | Parkin-mediated mitophagy | Activate | Protective | 22 |
| Methylation of mir-129-5P | Inhibiting Beclin-1 | Inhibit | Harmful | 23 |
| Mir-21 | PTEN/Akt | Inhibit | Harmful | 24-26 |
| Mir-210 | Silencing of ATG7 | Inhibit | Harmful | 27 |
| Mir‐153‐3p | Silencing of ATG5 | Inhibit | Harmful | 28 |
| Gefitinib | Inhibiting EGFR | Activate | Protective | 29 |
| Naringin | AMPK | Activate | Protective | 30 |
| Glucosamine | MTOR | Activate | Protective | 31 |
| 17β-estradiol | - | Activate | Protective | 32 |
| Resveratrol (RES) | PI3K Class1/Akt | Activate | Protective | 33,34 |
|  | AMPK/Sirt1 | Activate | Protective | 35 |
|  | - | Activate | Protective | 34 |
| Metformin | AMPK | Activate | Protective | 36 |
| Lactate | - | Activate | Harmful | 37 |
| Parathyroid hormone 1‑34 | MTOR | Activate | Protective | 38 |
| Apo866 (fk866) | - | Activate | Protective | 39 |
| Moracin M | PI3K Class1/Akt/mTOR | Activate | Protective | 40 |
| Berberine | - | Activate | Protective | 41 |
| Spermidine | - | Activate | Protective | 42 |
| Unbalanced Dynamic and Static Forces | - | Activate | Harmful | 43 |
| Compression | Ras/MEK/ERK/NRF1/ATG7 | Activate | Protective | 44,45 |
| Hyperosmotic stress | Ca^2+^-dependent AMPK/mTOR | Activate | Protective | 46 |
| Propionibacterium acnes (P. Acnes) | PI3K Class1/mTOR | Activate | Harmful | 47,48 |
| In AF | | | | |
| High glucose | ROS | Activate | Protective | 5,6 |
| Amino acid starvation | ER stress | Activate | Harmful | 43 |
| Serum and nutrient deprivation | - | Activate | - | 49 |
| Long-term serum starvation | Beclin-1-dependent | Activate | Harmful | 50 |
| transforming growth factor-β1 (TGF-β1) | PI3K Class1/Akt/mTOR & ERK1/2 | Inhibit | Protective | 50 |

**Reference**

1 Chen, K. *et al.* Autophagy Is a Protective Response to the Oxidative Damage to Endplate Chondrocytes in Intervertebral Disc: Implications for the Treatment of Degenerative Lumbar Disc. *Oxid Med Cell Longev* **2017**, 4041768, doi:10.1155/2017/4041768 (2017).

2 Zhang, Z. *et al.* Melatonin protects vertebral endplate chondrocytes against apoptosis and calcification via the Sirt1-autophagy pathway. *J Cell Mol Med* **23**, 177-193, doi:10.1111/jcmm.13903 (2019).

3 Xu, H. G. *et al.* Autophagy protects end plate chondrocytes from intermittent cyclic mechanical tension induced calcification. *Bone* **66**, 232-239, doi:10.1016/j.bone.2014.06.018 (2014).

4 Jiang, L. *et al.* Apoptosis, senescence, and autophagy in rat nucleus pulposus cells: Implications for diabetic intervertebral disc degeneration. *J Orthop Res* **31**, 692-702, doi:10.1002/jor.22289 (2013).

5 Kong, C. G., Park, J. B., Kim, M. S. & Park, E. Y. High glucose accelerates autophagy in adult rat intervertebral disc cells. *Asian Spine J* **8**, 543-548, doi:10.4184/asj.2014.8.5.543 (2014).

6 Park, E. Y. & Park, J. B. High glucose-induced oxidative stress promotes autophagy through mitochondrial damage in rat notochordal cells. *Int Orthop* **37**, 2507-2514, doi:10.1007/s00264-013-2037-8 (2013).

7 Bretin, A. *et al.* Activation of the EIF2AK4-EIF2A/eIF2alpha-ATF4 pathway triggers autophagy response to Crohn disease-associated adherent-invasive Escherichia coli infection. *Autophagy* **12**, 770-783, doi:10.1080/15548627.2016.1156823 (2016).

8 Chang, H. *et al.* Early-stage autophagy protects nucleus pulposus cells from glucose deprivation-induced degeneration via the p-eIF2alpha/ATF4 pathway. *Biomed Pharmacother* **89**, 529-535, doi:10.1016/j.biopha.2017.02.074 (2017).

9 Xiong, X. *et al.* Protective effect of p53 on the viability of intervertebral disc nucleus pulposus cells under low glucose condition. *Biochem Biophys Res Commun* **490**, 1414-1419, doi:10.1016/j.bbrc.2017.07.055 (2017).

10 Chen, J. W. *et al.* Hypoxia facilitates the survival of nucleus pulposus cells in serum deprivation by down-regulating excessive autophagy through restricting ROS generation. *Int J Biochem Cell Biol* **59**, 1-10, doi:10.1016/j.biocel.2014.11.009 (2015).

11 Yin, H. *et al.* The involvement of regulated in development and DNA damage response 1 (REDD1) in the pathogenesis of intervertebral disc degeneration. *Exp Cell Res* **372**, 188-197, doi:10.1016/j.yexcr.2018.10.001 (2018).

12 Choi, H. *et al.* Hypoxia promotes noncanonical autophagy in nucleus pulposus cells independent of MTOR and HIF1A signaling. *Autophagy* **12**, 1631-1646, doi:10.1080/15548627.2016.1192753 (2016).

13 Chen, J. W. *et al.* The responses of autophagy and apoptosis to oxidative stress in nucleus pulposus cells: implications for disc degeneration. *Cell Physiol Biochem* **34**, 1175-1189, doi:10.1159/000366330 (2014).

14 Wu, X. *et al.* IAPP modulates cellular autophagy, apoptosis, and extracellular matrix metabolism in human intervertebral disc cells. *Cell Death Discov* **3**, 16107, doi:10.1038/cddiscovery.2016.107 (2017).

15 Buchkovich, N. J., Yu, Y., Zampieri, C. A. & Alwine, J. C. The TORrid affairs of viruses: effects of mammalian DNA viruses on the PI3K-Akt-mTOR signalling pathway. *Nat Rev Microbiol* **6**, 266-275, doi:10.1038/nrmicro1855 (2008).

16 Miyazaki, S. *et al.* Recombinant human SIRT1 protects against nutrient deprivation-induced mitochondrial apoptosis through autophagy induction in human intervertebral disc nucleus pulposus cells. *Arthritis Res Ther* **17**, 253, doi:10.1186/s13075-015-0763-6 (2015).

17 Jiang, W. *et al.* SIRT1 protects against apoptosis by promoting autophagy in degenerative human disc nucleus pulposus cells. *Sci Rep* **4**, 7456, doi:10.1038/srep07456 (2014).

18 Chen, J. *et al.* Sirt6 overexpression suppresses senescence and apoptosis of nucleus pulposus cells by inducing autophagy in a model of intervertebral disc degeneration. *Cell Death Dis* **9**, 56, doi:10.1038/s41419-017-0085-5 (2018).

19 Tu, J. *et al.* Sestrin-Mediated Inhibition of Stress-Induced Intervertebral Disc Degradation Through the Enhancement of Autophagy. *Cell Physiol Biochem* **45**, 1940-1954, doi:10.1159/000487970 (2018).

20 Zheng, G. *et al.* TFEB protects nucleus pulposus cells against apoptosis and senescence via restoring autophagic flux. *Osteoarthritis Cartilage* **27**, 347-357, doi:10.1016/j.joca.2018.10.011 (2019).

21 Chen, L. *et al.* Protein kinase RNA-like ER kinase/eukaryotic translation initiation factor 2alpha pathway attenuates tumor necrosis factor alpha-induced apoptosis in nucleus pulposus cells by activating autophagy. *J Cell Physiol* **234**, 11631-11645, doi:10.1002/jcp.27820 (2019).

22 Shen, J. *et al.* IL-1beta induces apoptosis and autophagy via mitochondria pathway in human degenerative nucleus pulposus cells. *Sci Rep* **7**, 41067, doi:10.1038/srep41067 (2017).

23 Zhao, K. *et al.* Methylation of microRNA-129-5P modulates nucleus pulposus cell autophagy by targeting Beclin-1 in intervertebral disc degeneration. *Oncotarget* **8**, 86264-86276, doi:10.18632/oncotarget.21137 (2017).

24 Chen, J. H. *et al.* ATM-mediated PTEN phosphorylation promotes PTEN nuclear translocation and autophagy in response to DNA-damaging agents in cancer cells. *Autophagy* **11**, 239-252, doi:10.1080/15548627.2015.1009767 (2015).

25 Lin, H. *et al.* Mechanism of microRNA-21 regulating IL-6 inflammatory response and cell autophagy in intervertebral disc degeneration. *Exp Ther Med* **14**, 1441-1444, doi:10.3892/etm.2017.4637 (2017).

26 Wang, W. J. *et al.* MiR-21 promotes ECM degradation through inhibiting autophagy via the PTEN/akt/mTOR signaling pathway in human degenerated NP cells. *Biomed Pharmacother* **99**, 725-734, doi:10.1016/j.biopha.2018.01.154 (2018).

27 Wang, C. *et al.* MiR-210 facilitates ECM degradation by suppressing autophagy via silencing of ATG7 in human degenerated NP cells. *Biomed Pharmacother* **93**, 470-479, doi:10.1016/j.biopha.2017.06.048 (2017).

28 Wang, X. B., Wang, H., Long, H. Q., Li, D. Y. & Zheng, X. LINC00641 regulates autophagy and intervertebral disc degeneration by acting as a competitive endogenous RNA of miR-153-3p under nutrition deprivation stress. *J Cell Physiol* **234**, 7115-7127, doi:10.1002/jcp.27466 (2019).

29 Pan, Z. *et al.* Therapeutic effects of gefitinib-encapsulated thermosensitive injectable hydrogel in intervertebral disc degeneration. *Biomaterials* **160**, 56-68, doi:10.1016/j.biomaterials.2018.01.016 (2018).

30 Zhang, Z. *et al.* Therapeutic Potential of Naringin for Intervertebral Disc Degeneration: Involvement of Autophagy Against Oxidative Stress-Induced Apoptosis in Nucleus Pulposus Cells. *Am J Chin Med*, 1-20, doi:10.1142/S0192415X18500805 (2018).

31 Jiang, L., Jin, Y., Wang, H., Jiang, Y. & Dong, J. Glucosamine protects nucleus pulposus cells and induces autophagy via the mTOR-dependent pathway. *J Orthop Res* **32**, 1532-1542, doi:10.1002/jor.22699 (2014).

32 Ao, P. *et al.* 17beta-estradiol protects nucleus pulposus cells from serum deprivation-induced apoptosis and regulates expression of MMP-3 and MMP-13 through promotion of autophagy. *Biochem Biophys Res Commun* **503**, 791-797, doi:10.1016/j.bbrc.2018.06.077 (2018).

33 Gao, J., Zhang, Q. & Song, L. Resveratrol enhances matrix biosynthesis of nucleus pulposus cells through activating autophagy via the PI3K/Akt pathway under oxidative damage. *Biosci Rep* **38**, doi:10.1042/BSR20180544 (2018).

34 Zhang, B., Xu, L., Zhuo, N. & Shen, J. Resveratrol protects against mitochondrial dysfunction through autophagy activation in human nucleus pulposus cells. *Biochem Biophys Res Commun* **493**, 373-381, doi:10.1016/j.bbrc.2017.09.015 (2017).

35 Wang, X. H. *et al.* Resveratrol attenuated TNF-alpha-induced MMP-3 expression in human nucleus pulposus cells by activating autophagy via AMPK/SIRT1 signaling pathway. *Exp Biol Med (Maywood)* **241**, 848-853, doi:10.1177/1535370216637940 (2016).

36 Chen, D. *et al.* Metformin protects against apoptosis and senescence in nucleus pulposus cells and ameliorates disc degeneration in vivo. *Cell Death Dis* **7**, e2441, doi:10.1038/cddis.2016.334 (2016).

37 Wu, W. *et al.* Lactate down-regulates matrix systhesis and promotes apoptosis and autophagy in rat nucleus pulposus cells. *J Orthop Res* **32**, 253-261, doi:10.1002/jor.22503 (2014).

38 Wang, X. Y., Jiao, L. Y., He, J. L., Fu, Z. A. & Guo, R. J. Parathyroid hormone 134 inhibits senescence in rat nucleus pulposus cells by activating autophagy via the mTOR pathway. *Mol Med Rep* **18**, 2681-2688, doi:10.3892/mmr.2018.9229 (2018).

39 Shi, C. *et al.* Nicotinamide Phosphoribosyltransferase Inhibitor APO866 Prevents IL-1beta-Induced Human Nucleus Pulposus Cell Degeneration via Autophagy. *Cell Physiol Biochem* **49**, 2463-2482, doi:10.1159/000493843 (2018).

40 Guo, F., Zou, Y. & Zheng, Y. Moracin M inhibits lipopolysaccharide-induced inflammatory responses in nucleus pulposus cells via regulating PI3K/Akt/mTOR phosphorylation. *Int Immunopharmacol* **58**, 80-86, doi:10.1016/j.intimp.2018.03.015 (2018).

41 Chen, Y. *et al.* Berberine suppresses apoptosis and extracellular matrix (ECM) degradation in nucleus pulposus cells and ameliorates disc degeneration in a rodent model. *Int J Biol Sci* **14**, 682-692, doi:10.7150/ijbs.24081 (2018).

42 Zheng, Z. *et al.* Spermidine promotes nucleus pulposus autophagy as a protective mechanism against apoptosis and ameliorates disc degeneration. *J Cell Mol Med* **22**, 3086-3096, doi:10.1111/jcmm.13586 (2018).

43 Ye, W. *et al.* Increased macroautophagy in the pathological process of intervertebral disc degeneration in rats. *Connect Tissue Res* **54**, 22-28, doi:10.3109/03008207.2012.715702 (2013).

44 Li, S. *et al.* Autophagy attenuates compression-induced apoptosis of human nucleus pulposus cells via MEK/ERK/NRF1/Atg7 signaling pathways during intervertebral disc degeneration. *Exp Cell Res* **370**, 87-97, doi:10.1016/j.yexcr.2018.06.012 (2018).

45 Ma, K. G. *et al.* Autophagy is activated in compression-induced cell degeneration and is mediated by reactive oxygen species in nucleus pulposus cells exposed to compression. *Osteoarthritis Cartilage* **21**, 2030-2038, doi:10.1016/j.joca.2013.10.002 (2013).

46 Jiang, L. B. *et al.* Activation of autophagy via Ca(2+)-dependent AMPK/mTOR pathway in rat notochordal cells is a cellular adaptation under hyperosmotic stress. *Cell Cycle* **14**, 867-879, doi:10.1080/15384101.2015.1004946 (2015).

47 Lin, Y. *et al.* Propionibacterium acnes induces intervertebral disc degeneration by promoting nucleus pulposus cell apoptosis via the TLR2/JNK/mitochondrial-mediated pathway. *Emerg Microbes Infect* **7**, 1, doi:10.1038/s41426-017-0002-0 (2018).

48 Yuan, Y. *et al.* Histological Identification of Propionibacterium acnes in Nonpyogenic Degenerated Intervertebral Discs. *Biomed Res Int* **2017**, 6192935, doi:10.1155/2017/6192935 (2017).

49 Yurube, T. *et al.* Serum and nutrient deprivation increase autophagic flux in intervertebral disc annulus fibrosus cells: an in vitro experimental study. *Eur Spine J* **28**, 993-1004, doi:10.1007/s00586-019-05910-9 (2019).

50 Ni, B. B. *et al.* The effect of transforming growth factor beta1 on the crosstalk between autophagy and apoptosis in the annulus fibrosus cells under serum deprivation. *Cytokine* **70**, 87-96, doi:10.1016/j.cyto.2014.07.249 (2014).
